# An effective “three-in-one” screening assay for testing drug and nanoparticle toxicity in human endothelial cells

**DOI:** 10.1371/journal.pone.0206557

**Published:** 2018-10-31

**Authors:** Marcela Filipova, Oumsalama K. Elhelu, Silvia H. De Paoli, Zuzana Fremuntova, Tibor Mosko, Dusan Cmarko, Jan Simak, Karel Holada

**Affiliations:** 1 Institute of Immunology and Microbiology, First Faculty of Medicine, Charles University and General University Hospital in Prague, Prague, Czech Republic; 2 Department of Biological Models, Institute of Macromolecular Chemistry, Academy of Sciences of the Czech Republic, v.v.i., Prague, Czech Republic; 3 Center for Biologics Evaluation and Research, Food and Drug Administration, Silver Spring, Maryland, United States of America; 4 Institute of Biology and Medical Genetics, First Faculty of Medicine, Charles University and General University Hospital in Prague, Prague, Czech Republic; West Virginia University School of Medicine, UNITED STATES

## Abstract

Evaluating nanoparticle (NP) toxicity in human cell systems is a fundamental requirement for future NP biomedical applications. In this study, we have designed a screening assay for assessing different types of cell death induced by NPs in human umbilical vein endothelial cell (HUVEC) culture. This assay consists of WST-8, LDH and Hoechst 33342 staining, all performed in one well, which enables an evaluation of cell viability, necrosis and apoptosis, respectively, in the same cell sample. The 96-well format and automated processing of fluorescent images enhances the assay rapidity and reproducibility. After testing the assay functionality with agents that induced different types of cell death, we investigated the endothelial toxicity of superparamagnetic iron oxide nanoparticles (SPIONs, 8 nm), silica nanoparticles (SiNPs, 7–14 nm) and carboxylated multiwall carbon nanotubes (CNTCOOHs, 60 nm). Our results indicated that all the tested NP types induced decreases in cell viability after 24 hours at a concentration of 100 μg/ml. SPIONs caused the lowest toxicity in HUVECs. By contrast, SiNPs induced pronounced necrosis and apoptosis. A time course experiment showed the gradual toxic effect of all the tested NPs. CNTCOOHs inhibited tetrazolium derivatives at 100 μg/ml, causing false negative results from the WST-8 and LDH assay. In summary, our data demonstrate that the presented “three-in-one” screening assay is capable of evaluating NP toxicity effectively and reliably. Due to its simultaneous utilization of two different methods to assess cell viability, this assay is also capable of revealing, if NPs interfere with tetrazolium salts.

## Introduction

Different types of newly engineered nanomaterials are under promising development for various biomedical applications including diagnostic and therapeutic tools for treating many serious pathologies, such as cancer [1] or neurodegenerative diseases [2]. The versatile properties of nanoparticles (NPs) may help to overcome many problems related to the successful delivery of drugs into the site of the lesion. NPs have a high surface-to-volume ratio that enables the delivery of a large load of transported drug [3]. The small size of NPs prolongs their circulation in the blood [4, 5] and supports their accumulation at the tumor site [6]. However, in the case of toxic NPs prolonged NPs circulation can affect the endothelial cells of blood vessels more profoundly [7, 8]. The advantage of NPs is their almost endless spectrum of modifications that make them capable of aiming at the target of choice [9]. Commonly used alterations in NPs are functionalization by poly(ethyleneglycol), carboxylation, conjugation with lipids, peptides, proteins, enzymes, DNA or RNA, etc. [9–11]. These modifications lead to the availability of a large number of different NPs, which must be examined for their possible impact on human health. Screening the *in vitro* toxicity of the candidate NPs is the first essential step in preclinical evaluations of the safety of these nanomaterials [12]. The standard method for basic examinations of cell viability is based on a colorimetric technique that is dependent on tetrazolium salt MTT (3-(4,5-Dimethyl-2-thiazolyl)-2,5-diphenyl-2H tetrazolium bromide). The MTT is reduced in viable cells to insoluble formazan, which must be dissolved before measuring the absorbance [13]. Some NPs, such as carbon nanotubes (CNTs), can stabilize the structure of formazan crystals and thus lead to their insolubility in solvents [14]. For this reason, the MTT assay is inappropriate for testing certain NPs and for use in a high-throughput screening format. The caspase and Annexin V assays are frequently used to assess apoptosis. However, these assays require additional cell handling (i.e., detaching, washing and sometimes transferring), which may lead to cell damage. Other apoptosis assays, such as the comet assay and DNA laddering, are based on the evaluation of DNA damage detected by gel electrophoresis [15]. The comet assay was developed for testing NP toxicity in a high-throughput screening format [16], but as a standalone method, this assay does not provide information on necrotic cell death. Necrosis can be investigated with neutral red or trypan blue dyes that are incorporated or excluded from viable cells. Unlike neutral red, which binds to the membranes of lysosomes in viable cells [17], trypan blue is accumulated only in the nonviable counterparts [18]. In spite of its usefulness for daily laboratory routines, the trypan blue method usually requires the harvesting of cells by trypsin, and the neutral red must be extracted from the viable cells by acidified ethanol solution [17, 18]. These complications make them inconvenient for high throughput or multiple assay screenings.

To allow for the more efficient screening of NP endothelial cell toxicity, we combined three independent tests into one cell death screening assay, which is performed in endothelial cell culture without harvesting the cells. First, the viability assay is based on a new generation of tetrazolium salt WST-8 (2-(2-methoxy-4-nitrophenyl)-3-(4-nitrophenyl)-5-(2,4-disulfophenyl)-2H-tetrazolium, monosodium salt) that is highly water-soluble and thus more suitable for use in cell cultures [19]. Moreover, the negative net charge prevents WST-8 from entering the cells, and its reduction occurs through electron transport across the plasma membrane of viable cells [20]. Second, the cell membrane integrity and thus cell necrosis (including necroptosis and secondary necrosis arising from terminal stage of apoptosis [21]) is evaluated by lactate dehydrogenase (LDH) assay based on the colorimetric assessment of the LDH enzyme released from damaged cells into the media [22]. Third, apoptosis is determined using the DNA staining fluorescent dye Hoechst 33342. The proportion of apoptotic bodies is counted from the fluorescent images of fixed cells with a specifically designed automatic macro in ImageJ software. Furthermore, the use of Hoechst 33342 staining also validates and confirms information about the extent of live cells gained from the WST-8 assay and thus decreases the probability of false data. All assays are performed in sequence on the cells in the very same well, which gives information about the viability, necrosis and apoptosis of the same population of cells and thus contributes to the coherence, reliability and relevance of the results.

## Materials and methods

### Nanomaterials

Superparamagnetic Fe_3_O_4_ NPs (SPIONs, provided as a 3% aqueous suspension without organic stabilizers, average particle size of 8±3 nm, #PL-A-Fe3O4-10 m) and hydrophilic SiO_2_ NPs (SiNPs, provided as a fumed nanopowder, average particle size of 7–14 nm, #PL-SiOF-25g) were purchased from PlasmaChem (Berlin, Germany). Pristine multiwall carbon nanotubes (CNTs, average particle diameter of 60 nm, length of 1 μm–2 μm, purity >95%) were purchased from SES Research (#900–1280, Houston, TX) and carboxylated according to the section on preparing carboxylated CNTs. All the NP types were dissolved in the MilliQ water at a concentration of 4 mg/ml. The stock solutions were sonicated by using an ultrasonic micro-tip at 30% intensity for three 20-second pulses with 1-minute resting intervals on ice immediately before cell application (Dynatech Sonic Dismembrator, model 300, ARTEK, Farmingdale, NY, USA).

### Chemicals and reagents

(S)-(+)-camptothecin (#C9911), Triton X-100 (#X100), paraformaldehyde (#P6148), potassium phosphate monobasic (#P5655), sodium phosphate dibasic (#S5136), and dimethyl sulfoxide (DMSO, #D8418) were purchased from Sigma-Aldrich (Prague, Czech Republic); staurosporine (#3510A) was purchased from Santa Cruz Biotechnology (Heidelberg, Germany); Hoechst 33342 (#17530) was purchased from AAT Bioquest (Sunnyvale, CA, USA); and fetal bovine serum (#16000–036) was purchased from Thermo Fisher Scientific (Waltham, MA, USA). Hydrogen peroxide (30% solution, #23980–11000), potassium chloride (#60130G1000), sodium chloride (#71380G1000), isopropyl alcohol (#17510), nitric acid 65% (#18980) and sulfuric acid 96% (#20450) were obtained from Penta (Prague, Czech Republic).

### Preparation of carboxylated multiwall CNTs

The purity of the pristine multiwall CNTs was evaluated before [23]. The CNTs were carboxylated by stirring them in a concentrated H_2_SO_4_ and HNO_3_ solution at a 3:1 (v/v) ratio at 70°C for 24 hours. The removal of these acids from the functionalized CNTs (CNTCOOHs) was performed by filtering the CNTCOOHs across a PTFE membrane filter with a pore size of 0.1 μm (Omnipore Membrane Filter, Merck, Prague, Czech Republic). After being triple washed with MilliQ water, the CNTCOOH suspension was dialyzed against 5 liters of MilliQ water at room temperature (Spectra/Por 7 Dialysis Membrane, molecular weight cutoff of 1 kDa, Carl Roth, Karlsruhe, Germany). The water was exchanged three times every 12 hours.

### Characterization of nanoparticles

#### Zeta potential

The zeta potentials (ζ) of the NPs (with a final concentration of 10 μg/ml in MilliQ water) were measured with a Nano-ZS Zetasizer (Malvern Instruments, Malvern, UK). The ζ-Potential values were calculated from the electrophoretic mobilities (an average of three subsequent measurements, each of which consisted of 15–100 runs) using the Henry equation in the Smoluchowski approximation, or μ = εζ/η, where μ is the electrophoretic mobility, *η* is the solvent viscosity and ε is the dielectric constant of the solvent.

#### Dynamic light scattering

The hydrodynamic radius (Rh) of the SPIONs, SiNPs and CNTCOOHs was measured by dynamic light scattering (DLS) in a MilliQ water or culture medium (EGM-2, 2% FBS) using a Nano-ZS instrument (ZEN3600, Malvern, UK). The intensity of scattered light was detected at angle θ = 173° using a laser with a wavelength of 632.8 nm. DLS data were evaluated using the DTS (Nano) program. The values were the mean of at least four independent measurements.

#### Spectroscopy

Suspensions of each type of NP dissolved in MilliQ water (at a final concentration of 10 μg/ml) were measured on a spectrophotometer (Biospectrometer, Eppendorf, Ricany, Czech Republic) to collect absorption spectra over the wavelength range from 250 to 800 nm (S1 Fig).

#### Transmission electron microscopy

Transmission electron microscopy was used to visualize the structure of the nanoparticles. Three μl of NPs diluted in MilliQ water (at a final concentration of 10 μg/ml) were dropped on nickel or copper grids covered with formvar-carbon membrane and air-dried. The grids were examined without any staining at 80 kV in an FEI Morgagni TEM equipped with a CCD MegaView III camera or at 120 kV in a Tecnai G2 Sphera 20 TEM equipped with a Gatan USC 1000 slow scan CCD camera.

### Cell culture

Human umbilical vein endothelial cells (HUVECs) were obtained from Lonza (#C2519A, Lonza Group Ltd, Basel, Switzerland). In brief, the HUVECs were cultured in endothelial cell growth medium (EGM-2) containing 2% fetal bovine serum (FBS) and supplements (#CC-3162, Lonza Group Ltd, Basel, Switzerland) at 37°C in a humidified 5% CO_2_ atmosphere. After reaching 80% confluence, the cells were harvested with trypsin/EDTA, centrifuged at 220xg for 5 minutes and 5000 cells were seeded in 100 μl of fresh EGM-2 media per well in 96-well plates (Techno Plastic Products, Trasadingen, Switzerland). After 48 hours, the HUVECs were treated with NPs or specialized reagents for the required amount of time. For all the experiments, the cells were used at the 3^rd^ or 4^th^ passage.

### “Three-in-one” cell death screening (CDS) assay

#### LDH assay

All the parts of the CDS assay experiment were performed from the very same 96-well plate and were started by seeding HUVECs according to a previously described protocol. After 48 hours of incubation at 37°C in the 5% CO_2_ incubator, the cells were treated in 6-replicates with 200 μl of the desired concentration of NPs dissolved in media, positive control compounds, or negative controls (freshly added medium). The plate was incubated for 24 hours (dose-response experiments) or 0, 3, 6, 12 and 24 hours (time-response experiment) at 37°C in 5% CO_2_. Afterwards, the plate was centrifuged at 400xg and RT for 3 minutes, and 50 μl aliquots of the supernatant were transferred into a fresh 96-well plate. The LDH assay reaction mixture (50 μl) from the LDH Cytotoxicity Detection Kit (Takara Bio, Inc., Kusatsu, Japan) was added to the transferred aliquots and plate-incubated at 37°C for 30 minutes. The absorbance was measured at wavelengths of 490 nm and 660 nm with a microplate reader tempered at 37°C (Victor^3^ 1420–012 Multilabel Counter, Perkin Elmer, Akron, OH, USA). The absorbance of NPs measured at 490 nm was significant and falsely increased the signal from the LDH assay. To eliminate the contribution of the NPs to the absorbance at 490 nm, we measured their absorbance at 660 nm, where the absorbance of the LDH assay reaction mixture was minimal. The absorbance corresponding to the LDH activity was calculated using the following formula:

$$\begin{array}{c} A(\mathrm{LDH}{)}^{490}=A^{490}‑k\cdot A^{660}\\ k=\frac{A_{\mathrm{bckg}}^{490}}{A_{\mathrm{bckg}}^{660}} \end{array}$$

where A^490^ and A^660^ represent actual measured values and k is the coefficient calculated by measuring the absorbances of NPs measured in media with LDH reaction mixture, but without the cells.

#### WST-8 cell viability assay

This assay was performed in the same 96-well plate containing treated HUVECs from which the media aliquots were removed for the LDH assay. To each well containing the remaining 150 μl of media, 15 μl of the reagent from Cell Counting Kit-8 (Dojindo EU GmbH, Munich, Germany) was added and the plate was incubated at 37°C in a 5% CO_2_ atmosphere for 3 hours. Subsequently, 100 μl aliquots of the media with developed color were transferred into a new 96-well plate and the absorbance of each well was read at 450 nm and 660 nm using the microplate reader. The subtraction of the absorbance contribution of NPs to the WST-8 absorbance was calculated using the following formula:

$$\begin{array}{c} A(\mathrm{WST}‑8{)}^{450}=A^{450}‑k\cdot A^{660}\\ k=\frac{A_{\mathrm{bckg}}^{450}}{A_{\mathrm{bckg}}^{660}} \end{array}$$

Values of the cell viability are expressed as a percentage of the negative control value.

#### Fluorescent microscopy

The HUVECs remaining in the original 96-well plate were incubated with 20 μg/ml of Hoechst 33342 in PBS (26.8 mM KCl, 14.7 mM KH_2_PO_4_, 1.37 M NaCl, and 80.9 mM Na_2_HPO_4_, pH 7.4) at 37°C in the 5% CO_2_ incubator for 30 minutes. After staining, the cells were fixed with 2% paraformaldehyde and analyzed by fluorescence microscopy (FM) with excitation at 350 nm and emission at 450 nm. The fluorescence images were captured with an inverted fluorescent Olympus IX70 microscope (magnification 10×10; Olympus Europe, Hamburg, Germany) equipped with a ProgRes MFcool camera (Jenoptik AG, Jena, Germany). NIS-Elements BR3.1 software (Laboratory Imaging Ltd, Prague, Czech Republic) was used to acquire the fluorescence images. The total number of cell nuclei and apoptotic bodies was calculated by processing the images by the automated macro Counting_of_Nuclei or Counting_of_Apoptotic_Bodies specifically designed by our lab in the ImageJ 1.50b software, as described in detail in the S1–S3 Files. The data are expressed as a percentage of negative (cell nuclei) or positive (apoptotic bodies) control value.

### Interference experiment for CNTCOOH with LDH assay

The interference of the CNTCOOHs with the LDH assay was tested on media containing semi-confluent (70–80%) HUVECs in a 6-well plate (50 000 cells/well, 48 h) treated with 5 μM camptothecin, 1 μM staurosporine, 4 mM H_2_O_2_ and 0.1% Triton X-100, or non-treated (by adding fresh EGM-2 media) for 24 hours. The media containing LDH that was released from the cells were collected and centrifuged at 2000xg for 5 minutes at RT. Each supernatant was split into two new Eppendorf tubes. CNTCOOHs (at a final concentration of 100 μg/ml) were added to the first tube and the second tube served as a negative control. The tubes were incubated at 37°C in a CO_2_ incubator for 1 hour. At the end of the incubation, 50 μl aliquots of the supernatants were transferred to a 96-well plate filled with 50 μl of reaction mixture from the LDH Cytotoxicity Detection Kit, and the color developed at 37°C and 5% CO_2_ for 30 minutes. The absorbance was measured at wavelengths of 490 nm and 660 nm with a microplate reader tempered at 37°C. The data are presented as a percentage of the positive control value.

### Statistical analysis

A one-way ANOVA followed by a Dunnett’s post-test analysis were performed with GraphPad Prism software version 5.03 (GraphPad Software, San Diego, CA, USA). The data are presented as mean values (n = 3) ± standard error of the mean. Standard error means are indicated as error bars and differences are considered statistically significant when p < 0.05, and they are denoted with (*) in the graphs. The lethal concentration (LC_50_) values (with 95% confidence limits) of the tested NPs were determined with Finney’s Probit analysis method [24] as downloaded for Microsoft Excel software [25].

## Results

### Characterization of NPs

**Fig 1:**
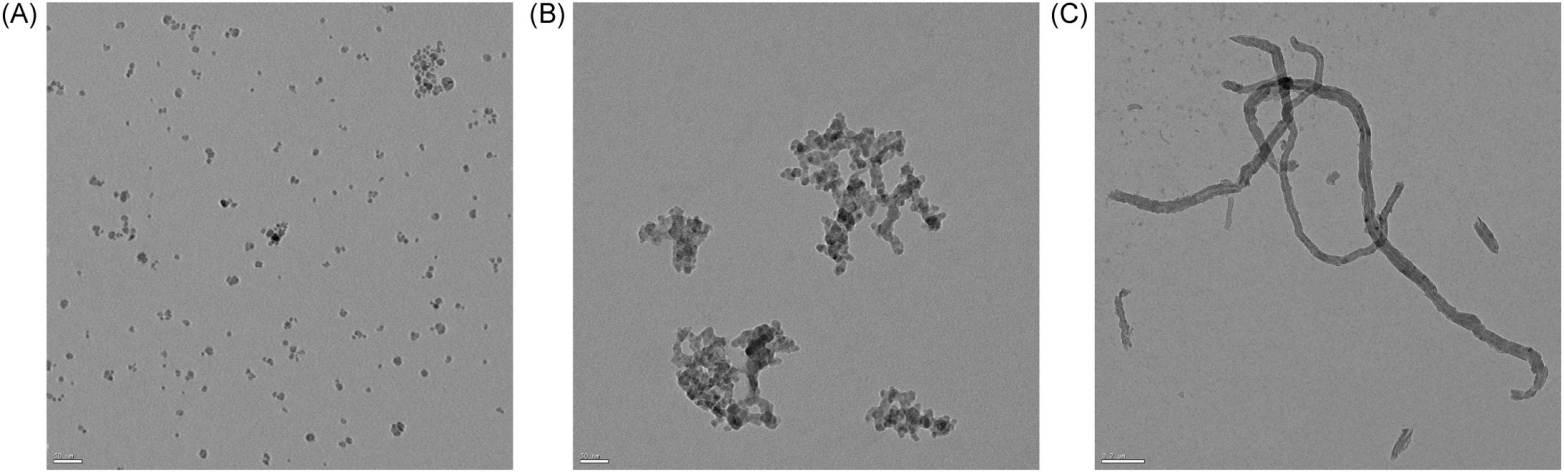
TEM images of NPs suspended in MilliQ water at a 10 μg/ml concentration. A) SPIONs, B) SiNPs and C) CNTCOOHs. Scale bars: 50 nm for SPIONs and SiNPs and 0.2 μm for CNTCOOHs.

The transmission electron microscopy (TEM) images of NPs dispersed in MilliQ water (Fig 1) showed the spheroid-hexagonal shape of the SPIONs (Fig 1A), the chain-like structure of SiNPs (Fig 1B) and rope-like morphology of CNTCOOHs (Fig 1C). Although the background of SPIONs and SiNPs is clean, the CNTCOOHs are surrounded with black dust that might correspond to amorphous carbon or residual carbon fragments. The agglomeration state of different NPs ranges from almost uniformly dispersed SPIONs to highly agglomerated SiNPs. Longer ropes of CNTCOOHs are individually dispersed or intermingled together. In the case of CNTCOOHs, the TEM images not only showed fibers of the expected lengths but also the shorter pieces of nanotubes, which may have originated from their production or from breaking during sonication immediately before cell treatment. It has been shown that sonication can cause the breakage of nanotubes [26].

The low value of CNTCOOH ζ-potential measurements (-52.5 ± 2.9 mV) shown in Table 1 correlates to the good physical stability of these NPs when dispersed in MilliQ water, unlike SPIONs (25.5 ± 2.1) and SiNPs (-28.4 ± 1.9) that are both over -30 mV, denoting a modest physical stability [27]. The negative value of the ζ-potential indicates the negative charge of SiNPs and CNTCOOHs, which is consistent with previously published information [28, 29]. SPIONs showed positive charges as indicated by the positive values of the ζ-potential. The size of the NPs on TEM images was consistent with the averages provided by the manufacturer, i.e., 8 ± 3 nm (SPIONs), 7–14 nm (SiNPs), 60–100 nm diameter and 1–2 μm length (CNTCOOHs). The hydrodynamic sizes of the NPs (Table 2) dispersed immediately in MilliQ water were 44, 210 and 219 nm for SPIONs, SiNPs and CNTCOOHs, respectively. Incubation of NPs for 24 hours in the culture medium including 2% FBS have coupled NPs together and increased the hydrodynamic size of NPs to 1081, 1140 and 634 nm (SPIONs, SiNPs and CNTCOOHs), respectively.

**Table 1:** Determination of NP stability in MilliQ water by ζ-potential measurement.

	Electrophoretic mobility (μm·cm·V^-1^·s^-1^)	Zeta potential (mV)
**SPION**	1.995 ± 0.2	25.5 ± 2.1
**SiNP**	-2.225 ± 0.1	-28.4 ± 1.9
**CNTCOOH**	-4.114 ± 0.2	-52.5 ± 2.9

Zeta potentials were measured by laser Doppler electrophoresis. Data are presented as the means ± SEM (n = 3).

**Table 2:** Hydrodynamic diameters of the NPs obtained by dynamic light-scattering (DLS).

	MilliQ water		Culture medium	
	Average size (nm)	PDI	Average size (nm)	PDI
**SPION (0 h)**	44.2 ± 4.7	0.22 ± 0.05	116 ± 8.2	0.32 ± 0.03
**SiNP (0 h)**	210 ± 32.3	0.18 ± 0.04	153 ± 23.1	0.31 ± 0.03
**CNTCOOH (0 h)**	219 ± 3.8	0.18 ± 0.01	261 ± 3.5	0.25 ± 0.01
**SPION (24 h)**	847 ± 44.3	0.48 ± 0.03	1081 ± 83.2	0.41 ± 0.06
**SiNP (24 h)**	340 ± 19.8	0.54 ± 0.02	1140 ± 105.1	0.60 ± 0.03
**CNTCOOH (24 h)**	295 ± 21.1	0.20 ± 0.01	634 ± 13.9	0.23 ±0.02

Data are presented as the means ± SEM (n = 4). **Abbreviations:** PDI = Polydispersity index; SPIONs, superparamagnetic iron oxide nanoparticles; SiNPs, silica nanoparticles; CNTCOOHs, carboxylated multiwalled carbon nanotubes.

### Validation of the cell death screening assay

**Fig 2:**
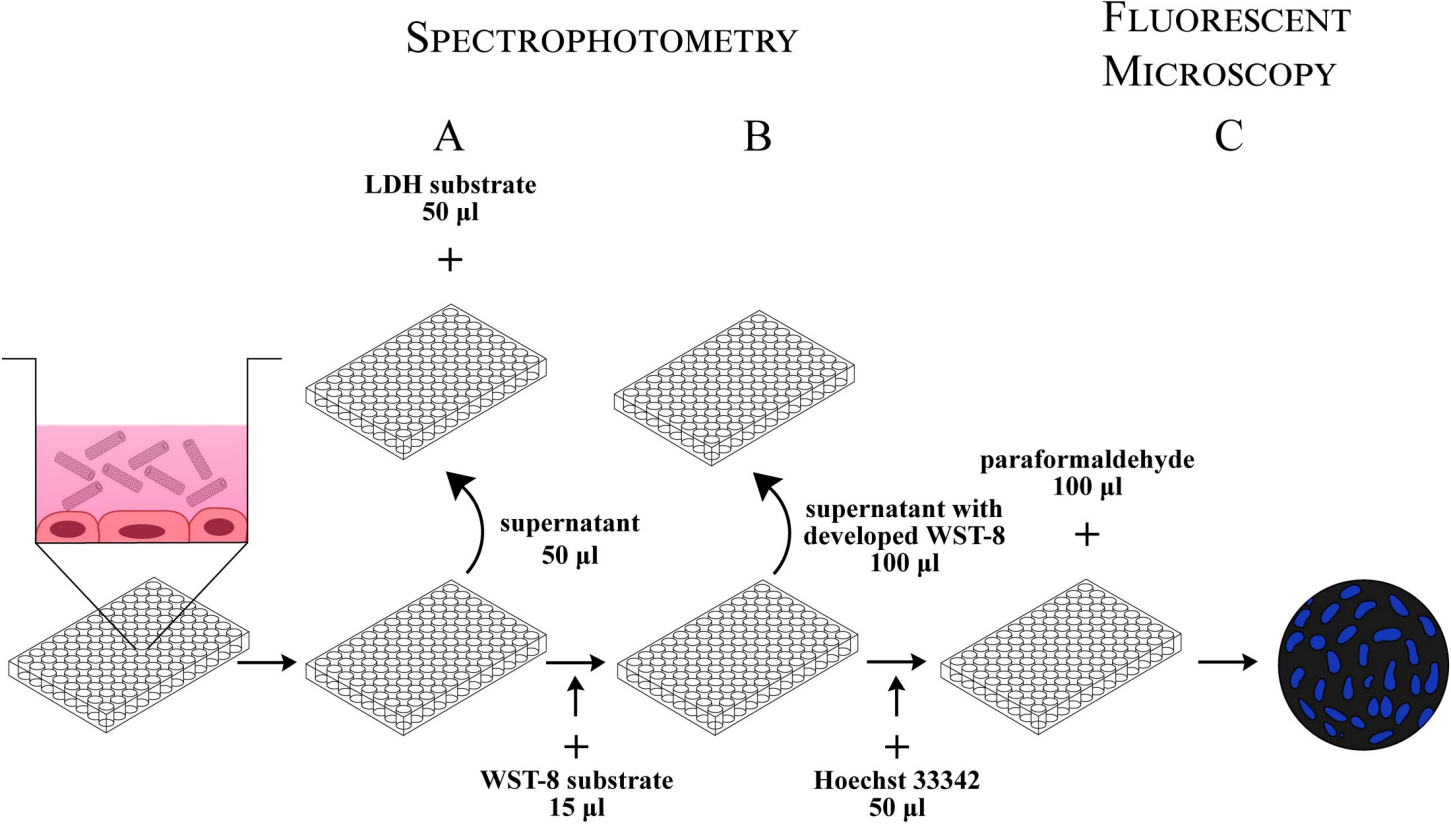
Workflow for the “three-in-one” cell death screening assay. HUVECs growing in the 96-well plate for 48 hours are exposed to NPs for 24 hours. Three types of cell death are evaluated simultaneously. A) Cell necrosis is measured spectrophotometrically after mixing an aliquot of cell supernatant with LDH substrate. B) Cell viability is assessed by adding WST-8 substrate to the cells. After three hours of incubation, aliquots of the reaction mixture are transferred into the new plate and measured spectrophotometrically. C) Cell apoptosis is detected after incubating the cells with Hoechst 33342 and fixing them with paraformaldehyde. Images captured under the inverted fluorescence microscope are computationally processed with the specially designed ImageJ macro.

To demonstrate the functionality of the CDS assay, we evaluated the time and dose responses of HUVECs to treatment with soluble agents that differed in their mechanism of cell death induction. Camptothecin and staurosporine were used to induce apoptosis [30–32], while hydrogen peroxide represented a positive control for necrotic cell death [33]. The cells were incubated with different concentrations of the agents for up to 24 hours, and the CDS assay was performed according to the schematic protocol depicted in Fig 2.

### Assessment of cell viability by WST-8 assay

**Fig 3:**
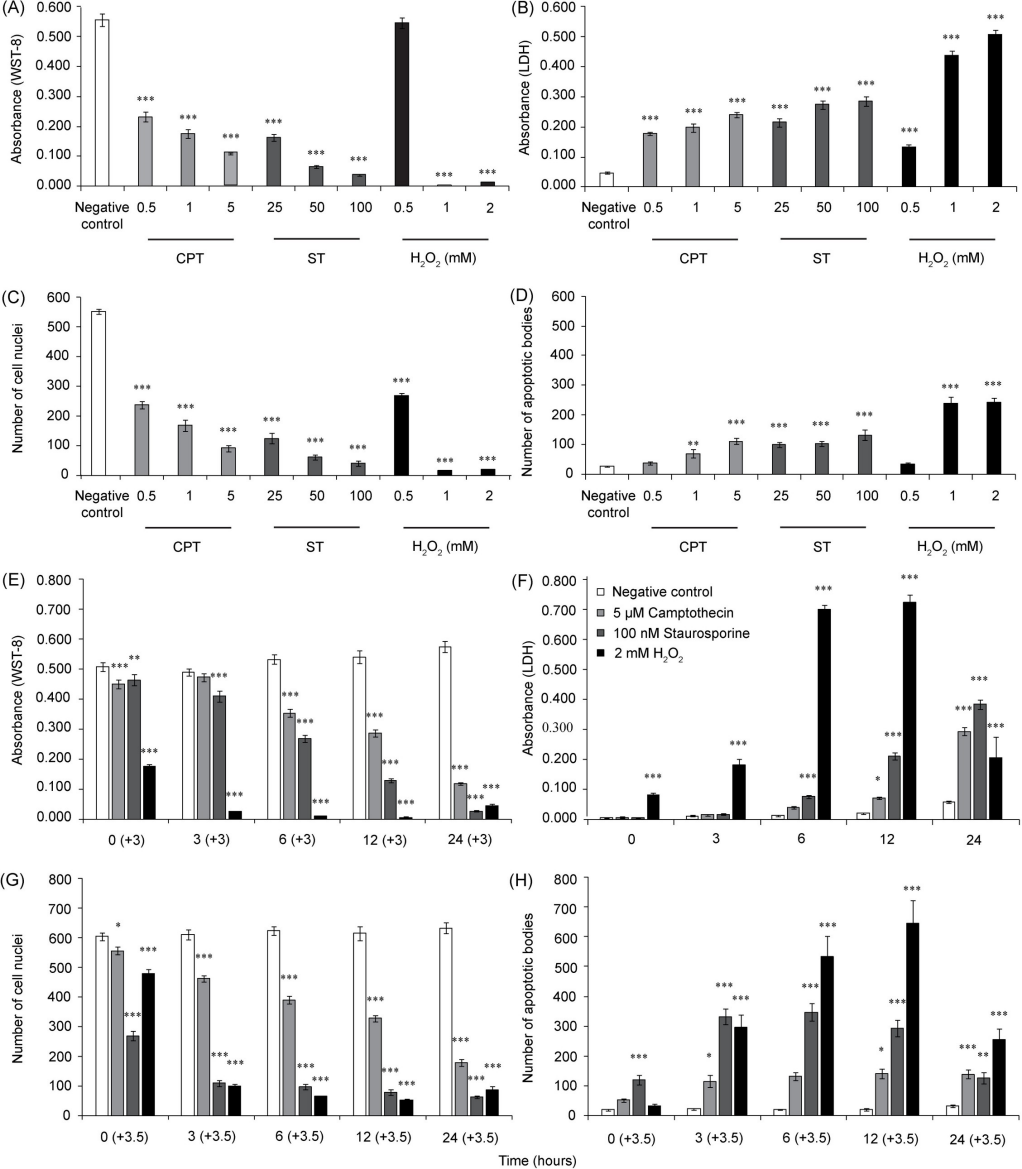
Evaluation of CDS assay performance. The HUVECs in the 96-well plate were treated with different concentrations of camptothecin, staurosporine or H_2_O_2_ for 24 hours (A–D), or with 5 μM camptothecin, 100 nM staurosporine or 2 mM H_2_O_2_ for 0, 3, 6, 12 and 24 hours (E–H). Cell viability was measured by WST-8 assay (A, E), and cell necrosis was evaluated by LDH assay (B, F). A count of the intact cell nuclei (C, G) and apoptotic bodies (D, H) was evaluated by ImageJ software after the cells were stained with Hoechst 33342. The WST-8 data and number of cell nuclei and apoptotic bodies were processed with 3h and 3.5h time difference, respectively. The data represent three independent experiments performed in 6-plicates. The bar graphs show the means ± SEM. Repeated measures were statistically tested by one-way ANOVA followed by Dunnett’s post-test. ***P<0.001, **P<0.01, and *P<0.05, versus the negative control.

Treatments with camptothecin and staurosporine (Fig 3A) showed dose-dependent cytotoxicity in the HUVECs. Camptothecin caused decreased cell viability to 42.0% (0.5 μM), 31.9% (1 μM) and 20.0% (5 μM) of the negative control after 24 hours (LC_50_ = 0.2 μM). Staurosporine induced a more substantial effect, resulting in the decline of cell viability to 29.4% (25 nM), 11.9% (50 nM) and 7.0% (100 nm) of the negative control after 24 hours (LC_50_ = 10.3 nM). The hydrogen peroxide at 0.5 mM did not influence the viability of the cells, in contrast to the 1 mM and 2 mM concentrations, which killed the cells completely within 24 hours (LC_50_ < 1 mM) (Fig 3A). In the time course experiment, the viability of the cells in the negative control was stable at 0, 3, 6, 12 and 24-hour measured time points. After exposing the HUVECs to 5 μM camptothecin, the cell viability gradually decreased from 88.6% (0 h) to 20.6% after 24 hours. The 100 nM staurosporine caused a more severe toxic effect with a sharply declining viability slope, from 91.3% (0 h) to 4.7% (24 hrs) vs the negative control. The effect of hydrogen peroxide on the cell viability was immediate, but we must keep in mind that the 0 h time point represents a 3 h cell incubation with the WST-8 substrate in the presence of the peroxide in the incubator, so there is enough time for fast-acting substances to damage the cells. The effect of hydrogen peroxide at the later time points resulted in the almost complete inhibition of the cell dehydrogenases (Fig 3E). In comparison, the effects of camptothecin and staurosporine became obvious only after 6 h (+3 h) of incubation with the cells, and the effect steadily progressed with time. Altogether, all the stimuli demonstrated a dose- and time-dependent decrease in HUVEC viability.

### Assessment of cell necrosis by LDH assay

An aliquot of media that was removed before adding the WST-8 reagent was used to assess the amount of LDH cytoplasmic enzyme released into the media. Both camptothecin and staurosporine induced a significant and concentration-dependent release of LDH (Fig 3B). The strongest effect was induced by 2 mM hydrogen peroxide, increasing the LDH level by approximately ten times in comparison to the negative control (Fig 3B). A time-dependent study of 5 μM camptothecin, 100 nM staurosporine and 2 mM hydrogen peroxide revealed differences in the LDH release dynamics among the tested agents (Fig 3F). Hydrogen peroxide evinced a small but significant LDH release even at 0 h. At the 12 h time point, all three stimuli induced a significant LDH release. The highest measured response was obtained from 2 mM hydrogen peroxide. After 12 hours of 2 mM H_2_O_2_ exposure, the release of LDH was elevated 36 times more compared to the negative control. Interestingly, the LDH level after 24 h of exposure was substantially lower than it was at 12 h, possibly due to the direct damaging effect of hydrogen peroxide on LDH activity [34]. The effect of camptothecin and staurosporine was gradual, with the highest LDH release occurring after 24 h of the treatment when it reached approximately 50% of the maximal effect elicited by the hydrogen peroxide (Fig 3F).

### Assessment of apoptosis and count of the intact nuclei with a fluorescence microscopy and semiautomatic macros

**Fig 4:**
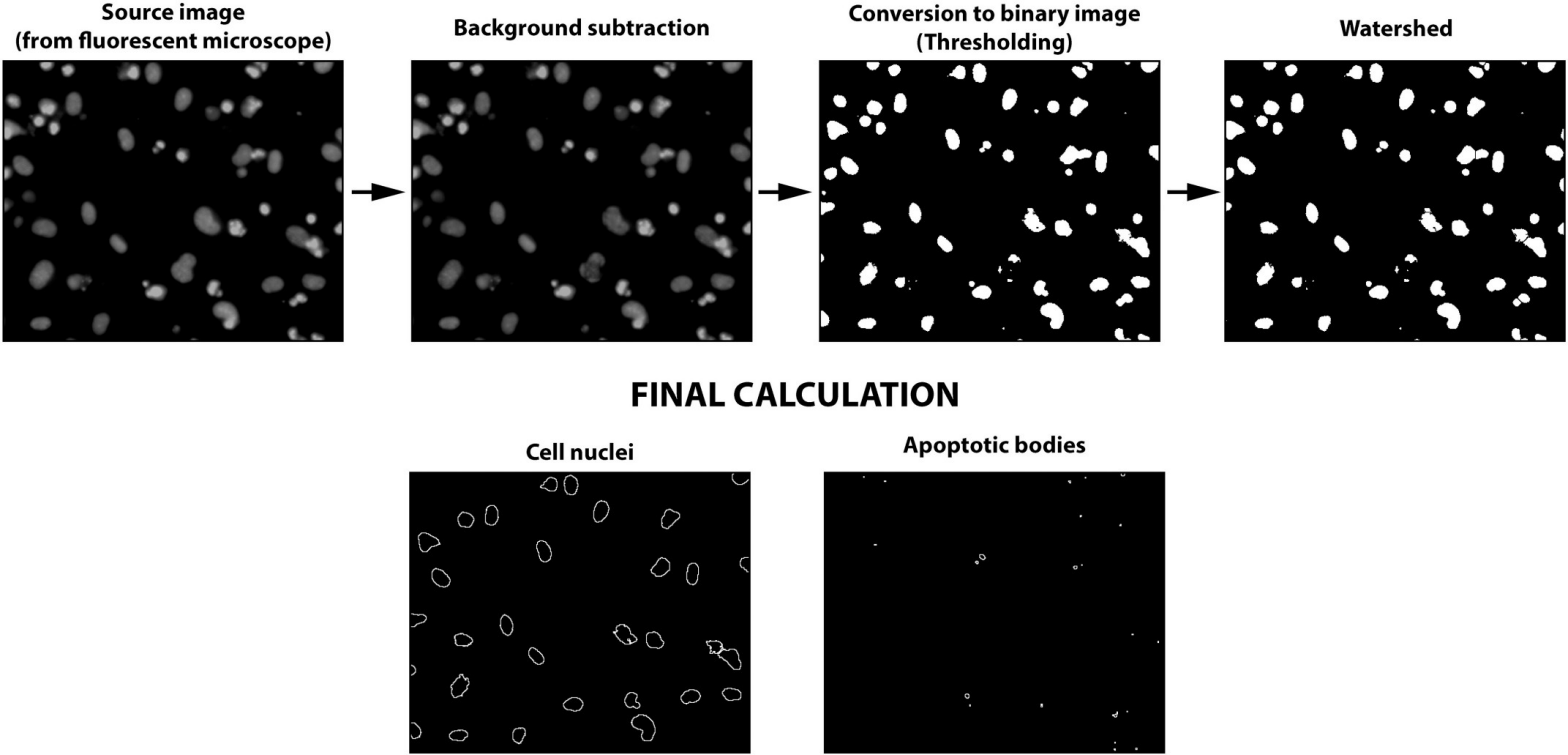
A schematic chart of the quantitative image analysis processed with ImageJ macros.

After incubating with WST-8, the cells were labeled with fluorescent Hoechst 33342 DNA dye and fixed, which enabled the investigators to visualize and subsequently distinguish intact cell nuclei from the apoptotic bodies of cells treated with different stimuli (Fig 4). The cell nuclei of nontreated HUVECs exhibited an oval shape with smoothly lined nuclei edges. The apoptotic cells exhibited chromatin condensation (pyknosis) and reduced nuclei sizes, finally leading to fragmentation into several small apoptotic bodies. The apoptotic bodies shine brightly under the fluorescence microscope, and captured images allow for the counting of bodies and intact nuclei. We exploited the open source ImageJ software (ImageJ 1.50b [35]) and designed cell-counting and apoptotic body-counting macros consisting of several steps (see the S1–S3 Files). We specifically set up two parameters, which helped to distinguish apoptotic bodies from cell nuclei and can be adjusted by user for size and brightness. The results from an analysis of representative pictures (Fig 4) show the counted cell nuclei and apoptotic bodies after applying the macros. The counted objects are depicted as a black circle. According to Syed Abdul Rahman et al., these intact cell nuclei can represent either viable cells or cells with impaired membranes in the early stage of necrosis [36], but at the same time, they have intact nuclei. In the dose-dependent experiments, the counted numbers of cell nuclei mirrored the viability of cells monitored by WST-8 assay (Fig 3C). In the time-dependent experiments, interesting discrepancies between the cell viability and nuclei counts were detected (Fig 3G). While camptothecin elicited a similar degree of time dependence in both measured factors, the effect of staurosporine on the cell nuclei count was more profound than it was on cell viability. Similarly, the numbers of apoptotic bodies displayed distinct kinetics, with the values being reached at different time points for different tested agents (Fig 3H). As with the WST-8 assay, we must keep in mind that the labeling of the cells and apoptotic bodies was performed after 3 h of cell incubation with the WST-8 substrate and occurred 30 minutes before fixation, so 3.5 h must be added to every monitored time point.

### Comparison of SiNPs, SPIONs and CNTCOOHs toxicity in HUVECs

The utility and functionality of the CDS assay for the evaluation of MP cytotoxicity was tested using 7–14 nm SiNPs, 8 nm SPIONs and 60 nm CNTCOOHs.

**Fig 5:**
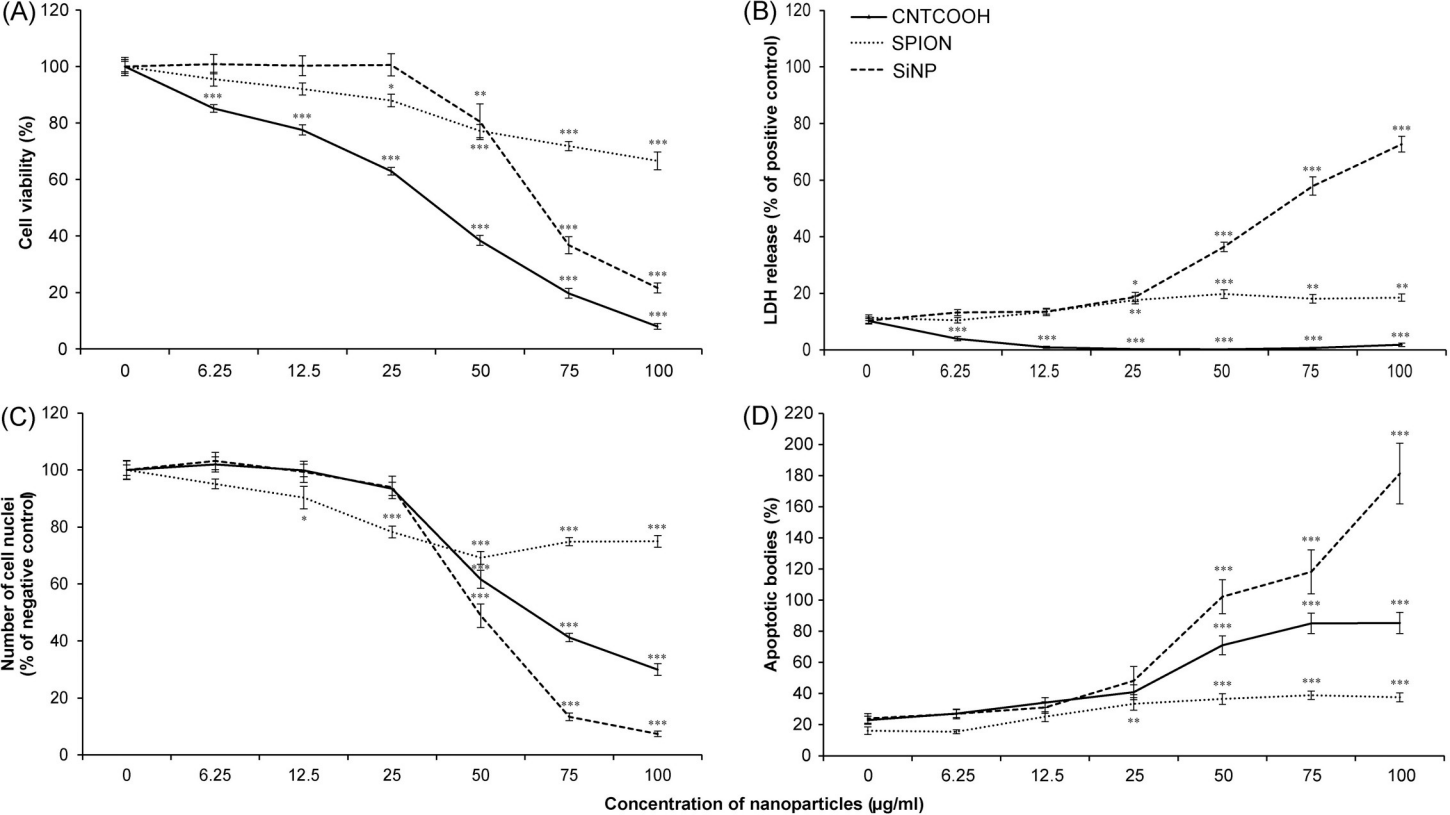
Dose-dependent toxicity of diverse NPs towards HUVECs as measured by CDS assay. The HUVECs in the 96-well plate were treated with 0–100 μg/ml of SiNP, SPION or CNTCOOH NPs for 24 hours. The cell viability was measured by WST-8 assay (A), the cell necrosis was gauged by LDH assay (B), and the number of intact cell nuclei (C) and number of apoptotic bodies (D) were counted by ImageJ software after staining the cells with Hoechst 33342. Each measurement was performed in 6-plicate, and the results (n = 3) are expressed as the means ± SEM as tested by one-way ANOVA followed by Dunnett’s test. ***P<0.001, **P<0.01, and *P<0.05, versus the negative control specific for each measurement.

SiNPs exhibited profound dose- and time-dependent cytotoxic effects in the HUVECs. The decrease in cell viability as measured by WST-8 assay started at 25 μg/ml and sharply continued to a concentration of 100 μg/ml after 24 h (Fig 5A). The LC_50_ as determined by WST-8 assay was 69.5 μg/ml. However, the LC_50_ calculated by counting intact cell nuclei (Fig 5C) showed that a lower concentration of 47.3 μg/ml is efficient for a 50% decrease in viable cells. The SiNPs concentration over 25 μg/ml caused a significant release of LDH (Fig 5B) and apoptotic bodies (Fig 5D), which were the highest of all the tested NPs.

**Fig 6:**
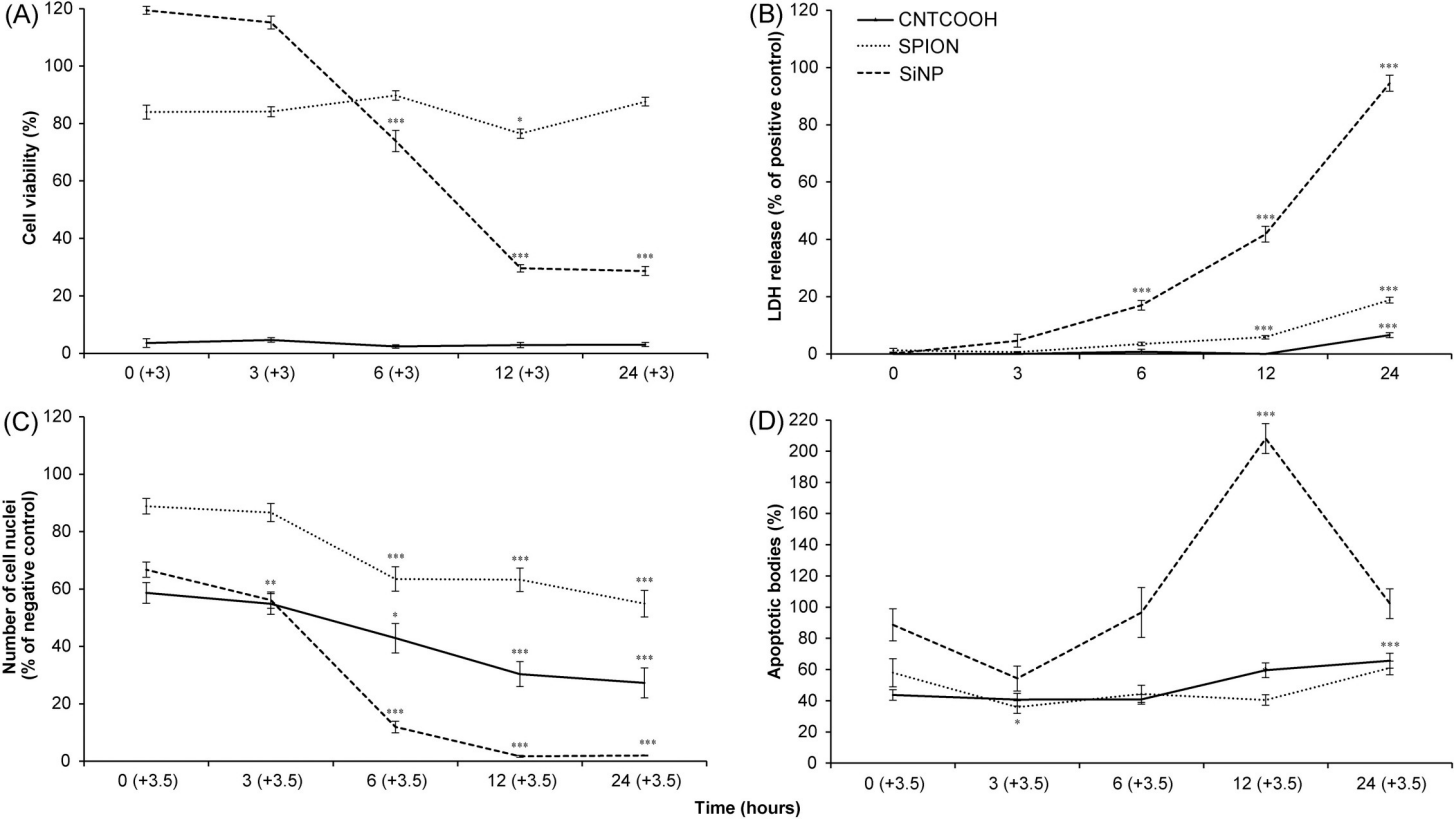
Time-response toxicity of different NPs towards HUVECs as measured by CDS assay. The HUVECs in the 96-well plate were treated with 100 μg/ml of SPION, SiNP and CNTCOOH NPs for 0–24 h. The cell viability was measured by WST-8 assay (A), the cell necrosis was determined by LDH assay (B), and the number of intact cell nuclei (C) and number of apoptotic bodies (D) were counted by ImageJ software after the cells were stained with Hoechst 33342. Each treatment was performed in 6-plicate and the results (n = 3) are expressed as the means ± SEM as tested by one-way ANOVA followed by Dunnett’s test. ***P<0.001, **P<0.01, and *P<0.05, versus the time point 0 hours.

The time-dependent experiment demonstrated that the cytotoxic effect of the SiNPs was rapid, causing a severe decline in cell viability and cell count within the first 6 hours of incubation (Fig 6A and 6C).

The concentration-dependent exposure of HUVECs to SPIONs led to a progressively slow decline in cell viability from 0 to 100 μg/ml, which is in accordance with the counting of the intact cell nuclei (Fig 5A and 5C). At the highest SPION concentration, the results demonstrated only a minute release of LDH and a modest increase in the count of apoptotic bodies (Fig 5B and 5D). While the cytotoxicity of SPIONs was so weak that the LC_50_ for this type of NPs could not be determined, still the 100 μg/ml of SPIONs caused significant decrease of cell viability measured by both WST-8 and counting of the cell nuclei (Fig 5A and 5C).

The results of the time-dependent experiments demonstrate a minor drop in the cell viability immediately after exposure to SPIONs (Fig 6A). This small decline is stable during the rest of the 24 hours and is significant in comparison to the negative control. By contrast, the number of the cell nuclei indicated a gradual development of a toxic effect as represented by a decrease in the number of intact cell nuclei, reaching 54.9% after 24 hours of treatment (Fig 6C). The SPIONs caused small release of LDH after 12 hours, which corresponds to small drop in cell viability in the same time point as measured by WST-8 assay (Fig 6B). The percentage of apoptotic bodies generated by SPIONs was close to the basal level in the negative control (Fig 6D).

The CNTCOOHs caused a profound dose-dependent decline in cell viability with a concentration of 100 μg/ml, lowering the WST-8 signal to 8.0% after 24 h (Fig 5A). The decrease in the number of intact nuclei was less intense, suggesting the possible interference of CNTs with the WST-8 assay (Fig 5C). The LC_50_ calculated by WST-8 assay was 27.7 μg/ml and differed from the LC_50_ determined by counting the cell nuclei at 66.1 μg/ml.

In addition, the time course experiment suggested that the toxic effect of CNTCOOHs on the cells measured by WST-8 assay was immediate (Fig 6A), while at the same time, the number of intact cell nuclei was close to 60% of the negative control (Fig 6C). While the number of cell nuclei decreased with the time of exposure, the absorbance of the WST-8 assay stayed the same. The number of apoptotic bodies showed a slow increase with time (Fig 6D) and their low numbers did correlate with the decreased amount of intact cell nuclei. Suspiciously, no release of LDH into the media was recorded (Figs 5B and 6B). Upon closer examination, the increasing concentration of CNTCOOH led to a decrease in the LDH assay signal (Fig 5B). It led us to the suspicion that CNTCOOHs might interfere with the results from the tetrazolium salts-based assays.

### Testing the interference of carboxylated multiwall carbon nanotubes by LDH assay

**Fig 7:**
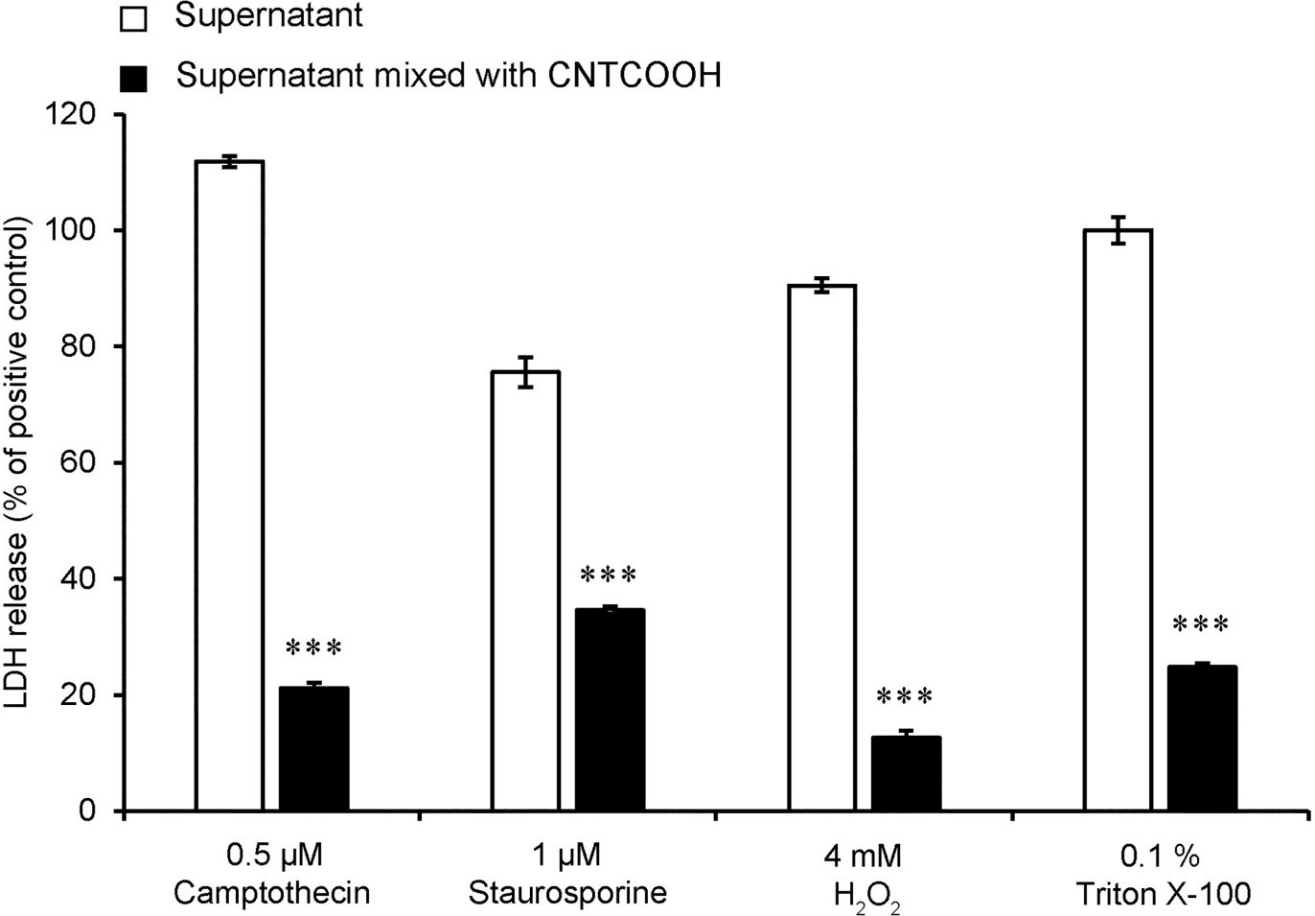
CNTCOOH NPs interfere with the LDH assay. HUVECs were treated with 0.5 μM camptothecin, 1 μM staurosporine, 4 mM H_2_O_2_ or 0.1% Triton X-100 for 24 hours to induce LDH release. After the treatment, the media were collected and centrifuged and the supernatant was divided into two aliquots. One aliquot was mixed with 100 μg/ml of CNTCOOHs and the other was left as a control. The presence of active LDH was measured colorimetrically. Data measured in 4-plicates (n = 3) are presented as the means ± SEM as tested by the two-tailed unpaired t-test, where ***P<0.001 versus the control.

The possible interference of CNTCOOHs with the LDH assay was tested on aliquots of media collected from HUVECs that were treated to induce LDH release. CNTCOOH addition significantly blocked the development of color, supporting the interference of the NPs with the assay (Fig 7). This interference can provide false negative results and incorrect evaluations of the cell necrosis caused by CNTCOOHs. From this result, it is obvious that the careful application of tetrazolium salt-based assays in combination with carbon-based nanomaterial belong to crucial points in the correct assessment of NPs *in vitro*.

## Discussion

The exceptional properties and diversity of newly developed nanomaterials brings not only great promises for use in biomedicine but also a number of challenges connected to the evaluation of their safety. The conventional approach to studying the toxicity of soluble compounds often displays unexpected limitations when utilized to study the interaction of NPs with the cells. The outcome of the NP-cell interactions can be greatly influenced by a number of factors such as the nature of the protein corona covering the NPs, or their agglomeration state [37]. These properties might be difficult to control and can produce stochastic results, which are difficult to interpret. In this study, we aimed to improve the effectiveness and robustness of the NP endothelial cell toxicity evaluation by combining three established methods aimed at the evaluation of the cell viability (WST-8), cell necrosis (LDH) and cell apoptosis (Hoechst staining) into a "three-in-one" cell death screening (CDS) assay. We believe that the assay is amendable also for other tightly adherent cell types forming monolayer. The primary advantage of performing three independent assays simultaneously on the cells in one well is the ability to correlate the obtained results directly without the danger of some unpredictable event affecting separate assays and complicating the interpretation of the data. Similar approach have been used also by Xia et al. [38]. The selection of the assays included in the CDS was intended to provide primary information about the type of cell death elicited by the tested NPs. As a general viability test, we utilized the last generation of tetrazolium salt, WST-8. In contrast to the MTT assay, the reduction of WST-8 is accomplished by electron transport on the surface of the cytoplasmic membrane, and the color product of the reaction is water soluble [20]. It has been shown that MTT-tetrazolium salt can provide false negative or false positive results due to the interaction with the compounds influencing the mitochondrial functions [39]. Probably, since the WST-8 is reduced through the trans-plasma membrane electron transport [40, 20], it is capable to provide reliable results also in the environment, where the MTT interfere with used compounds [40]. The WST-8 is also minimally toxic and the developed color can be measured in an aliquot of media without the need to harvest the cells, leaving them available for further evaluation. Similarly, the leakage of the LDH enzyme from necrotic cells with a damaged cytoplasmic membrane is estimated in an aliquot of media by measuring the LDH activity by using a colorimetric assay without affecting the cells. Finally, the cells are fluorescently labeled using Hoechst stain and fixed, allowing for the computational counting of the smooth nuclei from living cells and the small bright apoptotic bodies originating from the fragmentation of the cells undergoing apoptosis [41].

The functionality of the CDS assay was verified by evaluating the effect of apoptosis and necrosis-inducing agents, including camptothecin, staurosporine and hydrogen peroxide. All three stimuli caused a dose and time-dependent decrease in the cell viability and development of cell apoptosis and necrosis. Hydrogen peroxide was the most potent agent, not only in the extracellular release of LDH but also in the formation of apoptotic bodies. This finding is consistent with earlier research indicating the concentration-dependent role of hydrogen peroxide in triggering apoptosis or necrosis in cells [42]. As expected, the action of hydrogen peroxide was rapid while the toxic effect of the other compounds needed more time to develop. In general, the results obtained by separate assays correlated well, but on occasion, a discrepancy between the cell viability and number of intact cell nuclei, for example, was recorded. The technical factor contributing to such discrepancies may stem from the differences in the assays related to the length of the treatment time or from the possible interference with tested compounds as it has been shown previously with MTT-tetrazolium salt [40]. The LDH assay is performed in a separate plate so it reflects the amount of the released enzyme exactly at the time the media aliquot is transferred. In comparison, the development of the WST-8 assay requires a 3 h incubation with the treated cells, and only after the withdrawal of an aliquot of media for spectrophotometry are the remaining cells in the well stained with Hoechst stain for 0.5 h. Thus, the assays reflect the state of the cells, not at an identical time point, but rather different points over a 3.5 h examination window. While this reality may contribute to the complex interpretation of the CDS results, we believe that the guaranteed identity of the cell treatment greatly outweighs this technical deficiency.

To demonstrate the practicality of the CDS assay, we examined the toxicity of three NPs composed of different fundamental source materials, namely carbon, ferric oxide and silica oxide. All of the NPs used here are biomedically relevant and have been intensively researched for their unique physicochemical properties. For example, SPIONs display paramagnetic properties that can help to enhance the contrast of tumorous tissue using magnetic resonance imaging [43] or to deliver drugs by magnetic targeting [44]. Chemically modified silica nanoparticles (SiNPs) can deliver drugs directly into cancer cells [45, 46] and carbon nanotubes (CNTs) are under consideration for the thermal ablation of tumors [47, 48]. However, the exact same physicochemical properties cause different degrees of NP toxicity and interference with multiple assays such as MTT, MTS, LDH, neutral red or ^3^H-T radioactivity proliferation assays [49–52].

The DLS results showed that all studied NPs agglomerated in the culture media after 24 hrs incubation. PDI values demonstrated that all NPs made a highly polydisperse suspensions in water and in culture media. With high PDI values the obtained results on hydrodynamic diameters of NP agglomerates should be interpreted with a great caution.

The application of the CDS assay clearly demonstrated the differences in the toxicity of the studied NPs towards HUVECs. The 7–14 nm SiNPs showed the most pronounced decrease in cell viability, which was caused by the simultaneous development of necrosis and apoptosis. By contrast, the 8 nm SPIONs were the least, but still significantly toxic, and exhibited the best values among all the tested assays. Intriguing results were obtained by testing the toxicity of 60 nm multiwall CNTCOOH. Discrepancy between development of cell toxicity measured by WST-8 and trend in decreasing of intact cell nuclei number indicates inhibition effect of WST-8 with increasing concentration of CNTCOOH. Although we did not directly prove the interference of WST-8 with CNTCOOH, we did demonstrate the interference of the CNTCOOHs with the LDH assay in the absence of the cells. Thus we believe that our assay identified the issue of WST-8 interference invalidating the evaluation of cell viability. This was clearly demonstrated in time response experiment, in which high concentration of CNTCOOH completely blocked the development of WST-8 color while the number of cell nuclei enabled trustful assignment of CNTCOOH toxicity in HUVEC cells.

The inhibition potential of CNTs towards older tetrazolium-based assays is well-documented [49, 51, 53]. Regarding WST-8, it has been shown that carbon-based nanomaterials, including carbon nanotubes, belong to the natural acceptors of electrons [54]. WST-8 is extracellularly reduced across the plasma membrane via an intermediate electron acceptor [20]. It is possible that CNTCOOHs may sequester electrons as determined for the reduction of WST-8 and thus block the development of the color. Another option might be the direct sorption of the tetrazolium compounds on the surface of CNTCOOHs, lowering their effective concentration. A similar mechanism might be responsible for inhibiting the LDH colorimetric assay or alternatively, the direct interaction of CNTCOOHs with LDH may inhibit its enzymatic activity as was previously shown for carbon black NPs [55].

## Conclusions

In summary, we have developed a convenient, rapid, inexpensive and robust "three-in-one" cell death screening assay to evaluate NP endothelial cell toxicity. The combination of colorimetric WST-8 and LDH assays with the fluorescent labeling of cell nuclei and apoptotic bodies allows for the primary determination of the type of cell death and the confirmation of results obtained from the viability assay. The combined information provided by the CDS assay allows for the quick detection of possible confounding factors because there is a pronounced interference of CNTCOOHs with tetrazolium-based assays. The compact format of the CDS assay minimizes biological variability, lowers the amount of NPs needed for testing and offers the possibility of testing multiple samples in one plate. These features make the assay suitable for the high-throughput screening of different NPs and for testing the toxicity of the individual soluble components (drugs) used in NP-drug complexes. As such, the "three-in-one" CDS assay may facilitate and accelerate a primary decision about the cellular toxicity of newly developed NPs.

## Supporting information

S1 Fig Spectral contribution of CNTCOOH, SPION and SiNP NPs to results of colorimetric assays. Absorption spectra (1 cm path length) of CNTCOOH (C), SPION (D) and SiNP (E) NPs dissolved in MilliQ water to concentration 10 μg/ml were measured immediately after sonication (three 20s pulses with 1 minute pause interval incubated in ice bath). Thick black line shows absorbance measured at wavelength 660 nm contributing to increased background that is additionally subtracted from absorbance at 450 nm (dotted line) of WST-8 tetrazolium salt or absorbance at 490 nm (dashed line) of LDH. Absorption spectra of bare WST-8 (A) and LDH (B) without any NPs.
(TIF)

S1 File Macros description. Includes detailed macros description.
(DOCX)

S2 File Counting of nuclei. The macro for ImageJ software.
(DOCX)

S3 File Counting of apoptotic bodies. The macro for ImageJ software.
(DOCX)
