# Supplementary figures and images for: An effective “three-in-one” screening assay for testing drug and nanoparticle toxicity in human endothelial cells
Source: PLoS One. 2018 Oct 31;13(10):e0206557. doi: 10.1371/journal.pone.0206557 (PMC6209339; doi:10.1371/journal.pone.0206557)

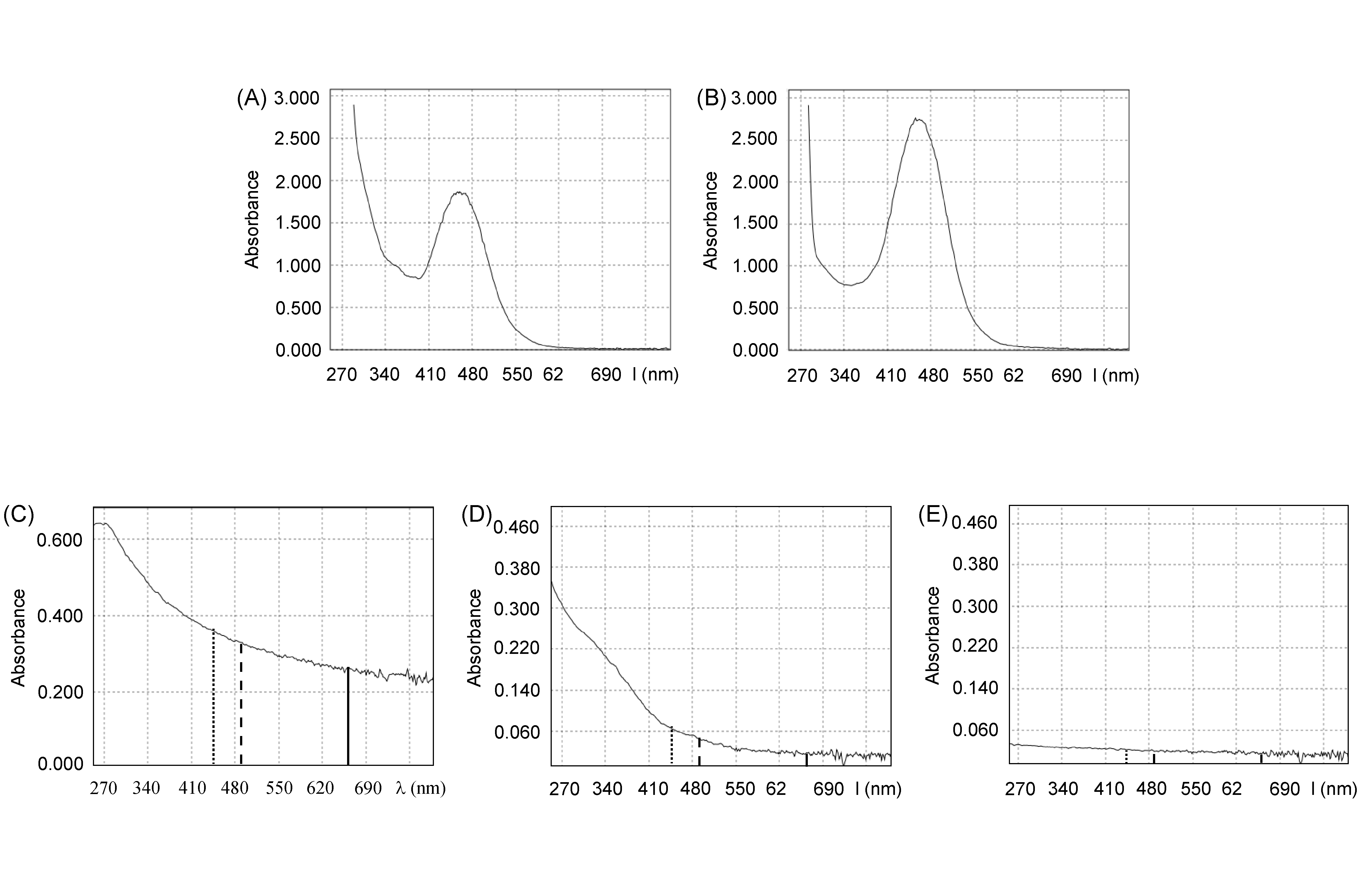

Supplement: S1 Fig — Absorption spectra (1 cm path length) of CNTCOOH (C), SPION (D) and SiNP (E) NPs dissolved in MilliQ water to concentration 10 μg/ml were measured immediately after sonication (three 20s pulses with 1 minute pause interval incubated in ice bath). Thick black line shows absorbance measured at wavelength 660 nm contributing to increased background that is additionally subtracted from absorbance at 450 nm (dotted line) of WST-8 tetrazolium salt or absorbance at 490 nm (dashed line) of LDH. Absorption spectra of bare WST-8 (A) and LDH (B) without any NPs. (TIF) [file pone.0206557.s001.tif]
