# Supplementary material for: An effective “three-in-one” screening assay for testing drug and nanoparticle toxicity in human endothelial cells
Source: PLoS One. 2018 Oct 31;13(10):e0206557. doi: 10.1371/journal.pone.0206557 (PMC6209339; doi:10.1371/journal.pone.0206557)
Supplement: S1 File — Includes detailed macros description. (DOCX) [file pone.0206557.s002.docx]

**Methods**

*Image thresholding*

Because the discrimination of apoptotic bodies by macro is dependent on two conditions, size and brightness intensity, the adjustment of background and set of the threshold was added in the case of counting_of_apoptotic_bodies macro. This improvement consists of following steps:

1) The uneven background illumination in the source images is smoothened with “Background subtraction” command. The standard implementation of the ImageJ employs the rolling ball algorithm[1]. The radius of the rolling ball should be set to the size of the smallest apoptotic bodies that is not part of the background but is still larger than nonspecific DNA fragments caused by too harsh treatments. For our purposes, the radius of the rolling ball was set on 12 (counting_of_nuclei macro) or 3 (counting_of_apoptotic_bodies macro).

2) For automatic counting of particles is demanded to convert the image to binary file that consist of 1’s and 0’s (i.e. black - 0 and white - 1). In the case of the counting_of_nuclei macro, it is solved with “convert to mask” command. But in the counting_of_apoptotic_bodies macro, the conversion to binary file is ensured with thresholding. The setting of threshold is important for the precise selection of object of interest by macro and it is possible to adjust the threshold range manually by the slider bars. In our images, the upper threshold was set to 114 and lower one to 255 with checked “dark background” command.

*Watershed*

To distinguish the overlapping cells or cells growing in close proximity is critical to use the Watershed algorithm[2] that work well with non-circular objects[3].

*Calculation of analyzed particles*

Final count of all objects that correctly complied highly described criteria is performed with “Analyze Particles” command in ImageJ. In the counting_of_nuclei macro, set up values were: size: 250 - 1500 pixels, circularity: 0 - 1, show: Outlines, Summarize, Exclude on edges, In situ show checked. Different values were set up in the case of counting_of_apoptotic_bodies macro: size: 0 - Infinity, circularity: 0 - 1, show: Outlines, Summarize, Exclude on edges, In situ show checked. The total number of detected objects might be advantageously exported to the Microsoft^®^ Excel^®^ software.

**References**

1. Sternberg SR. Biomedical image processing. Computer. 1983;16(1):22-34.

2. Vincent L, Soille P. Watersheds in digital spaces: an efficient algorithm based on immersion simulations. IEEE transactions on pattern analysis and machine intelligence. 1991;13(6):583-98.

3. Choudhry P. High-Throughput Method for Automated Colony and Cell Counting by Digital Image Analysis Based on Edge Detection. PLoS One. 2016;11(2):e0148469. doi: 10.1371/journal.pone.0148469. PubMed PMID: 26848849; PubMed Central PMCID: PMCPMC4746068.
