# Supplementary material for: An effective “three-in-one” screening assay for testing drug and nanoparticle toxicity in human endothelial cells
Source: PLoS One. 2018 Oct 31;13(10):e0206557. doi: 10.1371/journal.pone.0206557 (PMC6209339; doi:10.1371/journal.pone.0206557)
Supplement: S2 File — The macro for ImageJ software. (DOCX) [file pone.0206557.s003.docx]

**Counting_of_Nuclei**

source_path="C:\\Users\\%USERNAME%\\Desktop\\src_images\\";

output_path="C:\\Users\\%USERNAME%\\Desktop\\";

list=getFileList(source_path);

for(i=0;i<list.length;i++)

{

open(source_path+list[i]);

run("Subtract Background...", "rolling=12");

run("Convert to Mask");

run("Watershed");

run("Analyze Particles...", "size=250-1500 pixel show=Outlines exclude summarize in_situ");

saveAs("Tiff", output_path + list[i]);

close();

}

**Note:** It is recommended to double check correct username, name of the folder with images and other parts of the macros source and output path, otherwise macros will not run properly.
